# Association of age of adverse childhood experiences with thalamic volumes and post-traumatic stress disorder in adulthood

**DOI:** 10.3389/fnbeh.2023.1147686

**Published:** 2023-05-22

**Authors:** Nickelas Huffman, Chia-Hao Shih, Andrew S. Cotton, Terrence J. Lewis, Stephen Grider, John T. Wall, Xin Wang, Hong Xie

**Affiliations:** ^1^Department of Emergency Medicine, University of Toledo, Toledo, OH, United States; ^2^Department of Psychiatry, University of Toledo, Toledo, OH, United States; ^3^Department of Radiology, University of Toledo, Toledo, OH, United States; ^4^Department of Neurosciences, University of Toledo, Toledo, OH, United States

**Keywords:** thalamus, adverse childhood experiences (ACEs), post-traumatic stress disorder (PTSD), structural MRI, Childhood Age Range Stress Scale (CARSS)

## Abstract

**Background:**

Adverse childhood experiences (ACEs) have been linked to brain development and mental disorders, however, the impact of the age of occurrence of ACEs on thalamic volume and post-traumatic stress disorder (PTSD) after adult trauma remains unclear. This study assessed associations between ACEs at different ages to thalamic volumes and PTSD development following acute adult trauma.

**Methods:**

Seventy-nine adult trauma survivors were recruited immediately after trauma. Within 2 weeks of the traumatic event, participants completed the PTSD Checklist (PCL) to assess PTSD symptoms, the Childhood Trauma Questionnaire (CTQ) and Childhood Age Range Stress Scale (CARSS) to evaluate ACEs and perceived stress level at preschool (<6 years old) and school (6–13 years old) ages, and structural magnetic resonance imaging (sMRI) to measure thalamic volumes. Participants were divided into three groups: those who experienced no childhood trauma or stress (non-ACEs), those who experienced childhood trauma and stress onset at preschool ages (Presch-ACEs), and those who experienced childhood trauma and stress onset at school ages (Sch-ACEs). At 3 months, participants underwent PTSD symptom evaluation using the Clinician Administered PTSD Scale (CAPS).

**Results:**

Adult trauma survivors in the Presch-ACEs group had higher CTQ and CAPS scores. In addition, survivors in the Presch-ACEs group had smaller thalamic volume compared to survivors in the non-ACEs and Sch-ACEs groups. Furthermore, smaller thalamic volume moderated a positive association between post-trauma 2-week PCL and subsequent 3-month CAPS scores.

**Discussion:**

Earlier occurrence of ACEs was associated with smaller thalamic volume, which appears to moderate a positive association between early posttraumatic stress symptom severity and PTSD development after adult trauma. This raises the possibility that early occurrence of ACEs may impact thalamic structure, specifically a reduction in thalamic volume, and that smaller thalamic volume may contribute to susceptibility to PTSD development after adult trauma.

## Introduction

Post-traumatic stress disorder (PTSD) is recognized by post-traumatic stress symptoms (PTSS), including trauma re-experiencing, negative mood and cognition, avoidance, and hyperarousal symptom clusters. PTSD can be clinically diagnosed if the symptoms continue longer than one month after trauma (Calhoun et al., 2012; Jorge, 2015). PTSD is debilitating and can exert devastating impacts on affected individuals which also affect family, friends, and broader society (Hilton et al., 2019). According to the DSM-V, the prevalence of PTSD is nearly 7% (Weathers, 2017), however, factors for vulnerability to PTSD development after trauma exposure remain conjectural.

Adverse Childhood Experiences (ACEs) involve stressful or traumatic childhood events that occur before 18 years of age, such as abuse, neglect, parental separation, and other stresses (Gilgoff et al., 2020). ACEs are becoming an increasingly recognized concern that has medical and mental health consequences in adulthood (Petruccelli et al., 2019), including PTSD after adult trauma (Brewin et al., 2000; Xie et al., 2021). A prior study has shown that there is a 30% lifetime chance of developing PTSD in victims of childhood abuse or neglect, compared to a 20% chance in those who have experienced neither abuse nor neglect (Widom, 1999).

One potential mechanism underling the relationship between ACEs and PTSD is an effect of ACEs on brain structure. Associations between ACEs and abnormalities in the brain structure have been observed in the hippocampus, amygdala, and prefrontal cortex (Bremner et al., 1997; De Bellis et al., 2002; Hart and Rubia, 2012; Evans et al., 2016). The thalamus is a key structure in circuits that contribute to PTSS and mediate fear processing, memory, cognition, and sensation (LeDoux, 1986; Steriade and Llinas, 1988; Bergmann, 2008; Penzo et al., 2015; Beas et al., 2018). Thalamic structural and functional alterations have been indicated in over-consolidation of traumatic memory (Brewin, 2001; Suvak and Barrett, 2011), incorrect integration of trauma-related sensory inputs (Brewin, 2001), and inappropriate attention processing (Suvak and Barrett, 2011). ACEs due to physical abuse have been suggested to associate with smaller thalamic volume (Hanson et al., 2010), and smaller thalamic volume has been associated with impaired stress responses after traumatic events (De Bellis and Zisk, 2014). Additionally, smaller thalamic volume has been reported in chronic PTSD veterans (Cardenas et al., 2011). Smaller thalamic volume has also been associated with severe symptoms in police officers with PTSD (Shucard et al., 2012). However, existing work leaves unanswered questions with respect to brain and behavioral consequences of ACEs on PTSD development in adulthood.

There is little research examining the age of occurrence of ACEs and subsequent effects on stress and the brain. It is known that human brain development begins prenatally with peak growth ending during adolescence (Stiles and Jernigan, 2010). Specifically, brain volume nearly quadruples in size during preschool years and reaches roughly 90% of the final adult brain volume by age six (Reiss et al., 1996; Iwasaki et al., 1997; Courchesne et al., 2000; Paus et al., 2001; Lenroot and Giedd, 2006; Stiles and Jernigan, 2010). However, the time of occurrence of ACEs during brain development is rarely addressed. To our knowledge, only one study reported that ACEs during preschool ages (5.5 ± 0.8 years old) appeared to be associated with volume decreases in the insula, hippocampus, amygdala, subgenual cingulate, and caudate; whereas, ACEs which occurred during school ages (8.3 ± 1.2 years old) appeared to be associated with volume decreases only in the insula (Luby et al., 2019).

Our previous work on adult trauma suggests PTSS can emerge during early post-trauma weeks, remain present during subsequent early months, and contribute to the development of PTSD at post-trauma 3 months (Xie et al., 2018). We have also reported that in adult trauma survivors, ACEs were negatively associated with early post-trauma thalamic volume, and thalamic volume negatively associated with PTSS severity; at the same time, ACEs positively correlated with PTSS severity (Xie et al., 2021). These findings raise the possibility that ACEs may lead to structural volume decreases in the thalamus, which may contribute to increased PTSD development after adult trauma.

Pursuing this thinking, in the present study, we examined associations between the age of occurrence of ACEs, thalamic volume, and early PTSS progression after adult trauma. The design of the study included self-reported ACEs and PTSD Checklist-Stressor Specific for DSM-V (PCL) scores within 2 weeks post-trauma, magnetic resonance imaging (MRI) within 2 weeks post-trauma, and PTSD symptom evaluation using the Clinician-Administered PTSD Scale (CAPS) at 3 months post-trauma. Our study design assessed risks, associations, and moderation findings between these variables. Given our previous findings that early post-trauma thalamic volume was associated with both ACEs and early PTSS severity, we were particularly interested in elucidating whether there was evidence for the further possibility that early post-trauma PTSS severity, as indicated by 2-week PCL scores, and subsequent PTSD, as indicated by 3-month CAPs scores, were positively associated and, if so, if this association might be moderated by smaller thalamic volume.

## Materials and methods

### Subject enrollment

Seventy-nine adult trauma survivors (age 18–59, 21 males, 58 females) who were admitted to and discharged from the hospital Emergency Departments (ED) within 48 h after a traumatic exposure were recruited. Trauma types included motor vehicle collision (MVC) (*n* = 44), physical assault (*n* = 29), sexual assault (*n* = 4), or other traumatic events (*n* = 2). All subjects required immediate medical treatment in the ED after which they were discharged. The survivors were excluded when they: (1) had severe injuries [i.e., Abbreviated Injury Scale (AIS) > 2] (Gennarelli and Wodzin, 2006), which required surgery that precluded MRI scanning within the planned timeframe, (2) experienced MVC with low pain [Numeric Pain Rating Scale (NPRS) < 6] (Kahl and Cleland, 2005), (3) had indications or history of moderate or severe traumatic brain injury, (4) could not read or write English, (5) were diagnosed with severe neurological, psychiatric, or mental problems, (6) were influenced by alcohol or other substances at the time of trauma, and/or (7) had contraindications for MRI scans, such as pregnancy, claustrophobia, and/or ferrous materials within body tissues. All survivors in the study gave written informed consent. Consented subjects immediately completed the PCL questionnaire to evaluate post-trauma stress level (Blevins et al., 2015). The current study focuses on the neuro-mechanisms of PTSD symptom development, not PTSD prevalence. Based on previous works, higher acute stress symptoms can predict later PTSD symptom development (Brewin et al., 2003), therefore, only the survivors with a high PCL score (PCL score ≥ 28) were recruited. All study procedures were approved by the Institutional Review Board.

### Psychological assessments

Within two weeks (10.4 ± 4.3 days) after trauma, all consented survivors completed the self-report PCL survey to assess PTSS severity, and this test demonstrates good internal consistency and retest reliability (Conybeare et al., 2012). Two ACEs surveys were also completed at this time. The 28-item self-report Childhood Trauma Questionnaire (CTQ) was used to quantitatively and qualitatively assess ACEs severity throughout childhood up to 18 years old (Thombs et al., 2007). Studies have found the CTQ demonstrates high internal consistency and good test-retest reliability (Bernstein et al., 1994). CTQ evaluates five types of childhood maltreatments, including emotional, physical, and sexual abuse, and emotional and physical neglect. Along with numerical CTQ sub-scores and the total score from the five types of maltreatments, the CTQ survey also defines severity level for each type of maltreatment as none, low, moderate, and severe. The Childhood Age Range Stress Scale (CARSS) was used to assess perceived stress level during the preschool years (before age 6) and during the school years (ages 6–13). The survivors rated their levels of stress on a scale of 1–6 (1 = “Essentially stress-free”, 3 = “About average”, 6 = “The most stressful I’ve ever known”) for the two age ranges separately. Scores of 1–3 were considered no/low stress and scores of 4–6 were considered moderate/high stress in the present analyses. The CARSS questionnaire focuses on childhood stress level in different age ranges that are critical periods for brain development, while the CTQ measures trauma throughout all of childhood.

At 3 months post-trauma, an experienced clinical psychologist blinded to ACEs assessments and structural magnetic resonance imaging (sMRI) analyses interviewed study participants for PTSD symptom development using the CAPS, which demonstrates high internal consistency, interrater reliability, and good test-retest reliability (Weathers et al., 2018).

### Study groups

Based on the CARSS and CTQ scores, all survivors fell into one of three groups: (1) non-ACEs group (*n* = 21): none/low stress in both age ranges in CARSS and no type of maltreatment in CTQ, (2) Presch-ACEs group (*n* = 26): moderate/high stress across both preschool and school age ranges in CARSS, and at least one moderate/severe maltreatment in CTQ, or (3) Sch-ACEs group (*n* = 32): moderate/high stress only during school age, but not preschool age, and at least one moderate/severe maltreatment in CTQ. Subjects who had high stress in CARSS, but no/low maltreatment in CTQ, or vice versa, were excluded from analyses.

### Structural MRI (sMRI) acquisition and processing

The survivors were scanned within 2 weeks after adult trauma using a 3T General Electric Signa HDx MRI scanner (GE Healthcare, Chicago, IL, USA). A high-resolution T1-weighted brain sMRI image was obtained using a validated three-dimensional volume inversion recovery fast spoiled gradient recall echo protocol (repetition time = 7.9 ms, echo time = 3 ms, inversion time = 650 ms, field of view = 25.6 cm × 25.6 cm, matrix = 256 × 256, slice thickness = 1 mm, voxel dimensions = 1 mm × 1 mm × 1 mm 164 contiguous axial slices) (Xie et al., 2018). Review of sMRI images by a radiologist indicated no qualitative brain abnormalities. The left and right thalamic and intracranial volumes (ICV) were measured using automated FreeSurfer (version 6, RRID:SCR_001847)^1^. Measures of the thalamic volumes were based on three-dimensional whole-brain segmentation and labeling according to a probabilistic atlas (Fischl, 2012). FreeSurfer segmentation was visually verified by an inspector blinded to psychological assessments and CAPS scores.

### Statistical analyses

Ages and number of post-trauma days at which sMRI was taken were compared for the three groups with one-way ANOVA tests. Chi-Square tests were used to test sex (M/F) composition and type of trauma across the three groups. Univariate ANOVA analyses were used to assess differences in the CTQ, PCL, and CAPS scores across the three groups. Partial correlation tested the relationships of the CTQ score to CARSS score, and the early PCL score to the follow-up 3-month CAPS score with adjustments for age and sex. Skewness test was used to test thalamic volume distribution. The left and right thalamic volumes were compared across the three groups using univariate ANCOVA with controls for age, sex, and ICV. Finally, the above group comparisons were repeated to test the studied trauma type effect on the PTSS severity and thalamic volumes.

Based on our previously observed association between ACEs-related early post-trauma thalamic volume and PTSS (Xie et al., 2021), moderation analyses of potential thalamic volume influences on a possible association between early post-trauma PCL scores and subsequent CAPS scores was of particular interest in this study. A simple moderation model with controls for age, sex, and ICV was used to test whether the relationship between early PCL scores and follow-up 3-month CAPS scores might be contingent on left and/or right thalamic volume. Johnson-Neyman analysis was then used to identify left and right thalamic volume with significant moderating effect on the PCL and CAPS relationships as cut-off volume size to define small or large thalamus.

Statistical analyses were conducted using SPSS version 26 (IBM Corp., Armonk, NY) and the ‘PROCESS’ macro for SPSS (Hayes, 2018). Data are reported as mean ± standard deviation, with alpha = 0.05 as the significance level.

## Results

Across the three groups, there was no significant difference in age [*F*(2, 76) = 0.51; *p* = 0.603], female/male composition [χ^2^(79) = 3.933; *p* = 0.140], or type of trauma [χ^2^(78) = 4.362; *p* = 0.628] (Table 1).

**TABLE 1:** Demographic.

	non-ACE	Presch-ACE	Sch-ACE
Subjects (F/M)	21 (12/9)	26 (21/5)	32 (25/7)
Age (year)	33.5 ± 11.9	35.1 ± 10.3	32.3 ± 9.4
sMRI taken since trauma (day)	10.6 ± 3.3	10.3 ± 4.5	10.3 ± 4.7
**Trauma type**			
MVC	14	12	18
Physical assault	5	12	12
Sexual assault	2	1	1
Other	0	1	1

### Associations of CTQ with CARSS scores and CTQ score comparisons across ACEs groups

**FIGURE 1:**
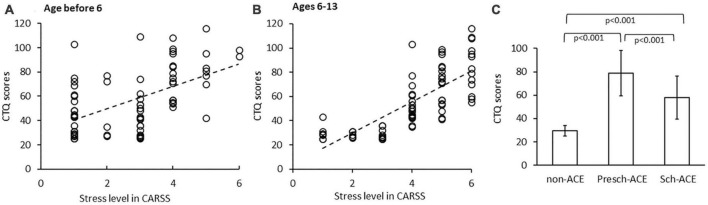
**(A)** Significant positive correlation between CTQ scores and CARSS scores before age 6. **(B)** Significant positive correlation between CTQ scores and CARSS scores in ages 6–13. **(C)** CTQ scores significantly differed across all 3 groups.

Positive correlations between CTQ scores and CARSS scores were shown in both Presch-ACEs and Sch-ACEs groups (*r* = 0.538, *p* < 0.001 and *r* = 0.732, *p* < 0.001, respectively, Figures 1A, B), suggesting the two measures in the current study used to assess ACEs were consistent.

Overall group comparison indicated a significant difference in CTQ scores among the three groups [*F*(2, 68) = 48.854, *p* < 0.001, partial η^2^ = 0.590]. *Post-hoc* tests indicated CTQ scores were significantly higher in both the Presch-ACEs and Sch-ACEs groups compared to the non-ACEs group (both *p* < 0.001). CTQ scores were also significantly higher in the Presch-ACEs group compared to the Sch-ACEs group (*p* < 0.001) (Table 2 and Figure 1C).

**TABLE 2:** Behavioral and brain measures.

	non-ACE	Presch-ACE	Sch-ACE
CTQ score	29.3 ± 4.5	78.9 ± 19.4*^†^	57.9 ± 18.4*
PCL score at 2 weeks	46.8 ± 12.7	56.1 ± 12.2	51.9 ± 17.3
CAPS score at 3 months	16.4 ± 13.6	27.9 ± 16.3*^†^	18.5 ± 12.5
**Thalamic volume (mm^3^)**			
Left	7523.4 ± 923.8	6751.3 ± 552.3*^†^	7122.8 ± 920.3
Right	6953.2 ± 816.1	6379.2 ± 470.4*^†^	6638.8 ± 834.7

*p < 0.05 when compared to non-ACE. ^†^*p* < 0.05 when compared to Sch-ACE, when controlling age and sex, and also controlling ICV in thalamic volume comparison.

### PCL and CAPS score group comparisons and correlations

**FIGURE 2:**
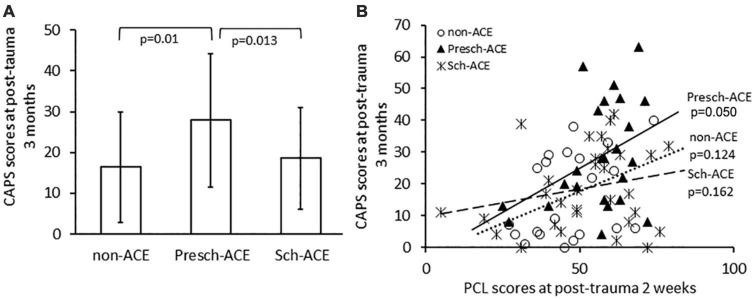
**(A)** CAPS scores significantly differed for Presch-ACEs vs. both non-ACEs and Sch-ACEs groups. **(B)** Significant positive correlation between PCL and CAPS scores in the Presch-ACEs group, but not the non-ACEs and Sch-ACEs groups.

Post-traumatic stress symptoms severity, which was assessed by PCL scores taken within 2 weeks post-trauma, was not significantly different across all groups [*F*(2, 71) = 2.209, *p* = 0.117, partial η^2^ = 0.059]. However, there was a significant difference in CAPS scores at post-trauma 3 months across the three groups [*F*(2,74) = 4.513, *p* = 0.014, partial η^2^ = 0.109]. CAPS scores were significantly higher in the Presch-ACEs, compared to both the non-ACEs (*p* = 0.01) and Sch-ACEs groups (*p* = 0.013). CAPS scores for the Sch-ACEs and non-ACEs groups did not significantly differ (*p* = 0.677, Table 2 and Figure 2A). When trauma type was applied as a covariant factor in analyses, the significant result in CAPS score comparison was maintained [*F*(2,71) = 4.145, *p* = 0.020, partial η^2^ = 0.105].

PCL scores within 2 weeks after trauma were positively correlated with CAPS scores at post-trauma 3 months for all survivors [*r*(72) = 0.376, *p* = 0.001]. Further analyses indicated a significant positive correlation between PCL and CAPS scores in the Presch-ACEs group [*r*(20) = 0.423, *p* = 0.050], but not in the non-ACEs [*r*(17) = 0.365, *p* = 0.124] or Sch-ACEs [*r*(27) = 0.267, *p* = 0.162] group (Figure 2B).

### Thalamic volume comparisons across groups

**FIGURE 3:**
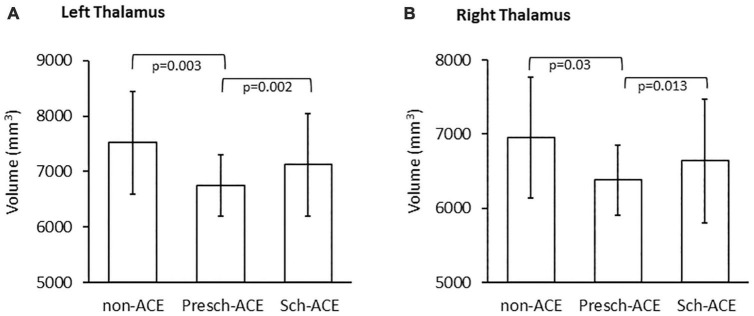
**(A)** Significant difference in left thalamic volume between the Presch-ACEs vs both non-ACEs and Sch-ACEs groups. **(B)** Significant difference in right thalamic volume between the Presch-ACEs vs both non-ACEs and Sch-ACEs groups.

Skewness tests showed that the left and right thalamic volumes had normal distribution (Skewness = 0.351, SE = 0.271 and Skewness = 0.486, SE = 0.271, respectively). Left thalamic volume significantly differed across the three groups [*F*(2,73) = 6.823, *p* = 0.002, partial η^2^ = 0.157]. *Post hoc* tests indicated that left thalamic volume was significantly smaller in the Presch-ACEs (*p* = 0.003), but not Sch-ACEs (*p* = 0.899) group, when compared to the non-ACEs group. Left thalamic volume was also significantly smaller in the Presch-ACEs group when compared to the Sch-ACEs group (*p* = 0.002) (Table 2 and Figure 3A).

Similar results were seen for right thalamic volume. There was a significant difference in right thalamic volume across the three groups [*F*(2,73) = 3.866, *p* = 0.025, partial η^2^ = 0.096]. Right thalamic volume was significantly smaller in the Presch-ACEs (*p* = 0.03), but not the Sch-ACEs (*p* = 0.949) group, when compared to the non-ACEs group. Right thalamic volume was also significantly smaller in the Presch-ACEs group compared to the Sch-ACEs group (*p* = 0.013) (Table 2 and Figure 3B).

When trauma type was applied as a covariant factor in the cross-group comparison, significant results in both left and right thalamic volume analyses were maintained [left: *F*(2,70) = 6.654, *p* = 0.002, partial η^2^ = 0.160; right: *F*(2,70) = 4.219, *p* = 0.019, partial η^2^ = 0.108].

### Moderating effects of the left and right thalamus on the positive association between post-trauma 2-week PCL scores and 3-month CAPS scores

**FIGURE 4:**
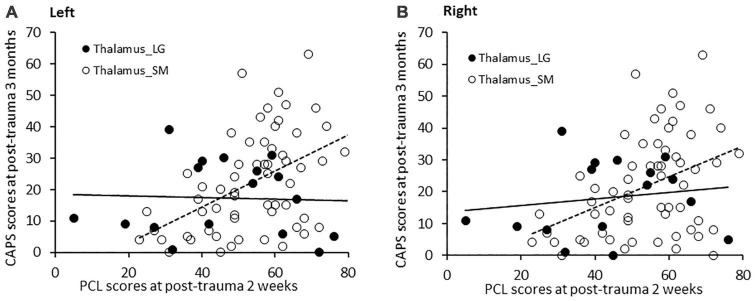
**(A)** PCL scores at post-trauma 2 weeks significantly positively correlated with CAPS scores at post-trauma 3 months in patients with a small left thalamic volume (<7,853 mm^3^), but not in patients with a large left thalamic volume. **(B)** Similarly, PCL scores at post-trauma 2 weeks significantly positively associated with CAPS scores at post-trauma 3 months in patients with a small right thalamic volume (<7,230 mm^3^), but not in patients with a large right thalamic volume.

With age, sex, and ICV controlled, PCL scores at post-trauma 2 weeks significantly positively associated with CAPS scores at post-trauma 3 months for the three groups (β = 2.673, SE = 0.966, *t* = 2.767, *p* = 0.007). Left thalamic volume moderated this association (β = −0.309, SE = 0.130, *t* = −2.381, *p* = 0.020). Johnson-Neyman analysis further indicated that PCL scores significantly positively associated with CAPS scores when left thalamic volumes were smaller than 7853 mm^3^ as seen in 77.6% of subjects; whereas, in contrast, PCL and CAPS scores were not significantly associated when left thalamic volumes were larger than 7853 mm^3^ (see respectively Thalamus_SM and Thalamus_LG in Figure 4A).

Similarly, moderation analysis for the right thalamus, with age, sex, and ICV controlled, revealed that PCL scores at post-trauma 2 weeks significantly positively associated with CAPS scores at 3 months across the three groups, (β = 3.006, SE = 1.085, *t* = 2.771, *p* = 0.007) and that right thalamic volume moderated this association (β = −0.381, SE = 0.158, *t* = −2.420, *p* = 0.018). Johnson-Neyman analysis further indicated that PCL scores significantly positively associated with CAPS scores when right thalamic volumes were smaller than 7230 mm^3^ as seen in 78.8% of subjects; whereas, in contrast, PCL and CAPS scores were not significantly associated when right thalamic volumes were larger than 7230 mm^3^ (see respectively Thalamus_SM and Thalamus_LG in Figure 4B).

These findings indicate that associations between early PCL scores and later CAPS scores after adult trauma were related to left and right thalamic volumes.

## Discussion

### Present findings

To the best of our knowledge, this is the first attempt to assess potential associations between age of occurrence of ACEs, and thalamic volume, PTSS, and PTSD over early times after subsequent adult trauma.

Adult trauma survivors with a history of preschool ACEs had higher CTQ and post-trauma 3-month CAPS scores when compared to survivors with either a later onset of ACEs or no history of ACEs. Smaller left and right thalamic volumes were seen in the trauma survivors with preschool ACEs compared to the survivors with later onset of ACEs or no ACEs history. Finally, thalamic volume moderated a positive relationship between PCL scores at post-trauma 2 weeks and CAPS scores at post-trauma 3 months.

### Associations between age of occurrence of ACEs and PTSS after subsequent adult trauma

Multiple factors likely affect PTSS after adult trauma. For example, acute PTSS can be influenced by emotional and dissociative responses to the traumatic event (Brewin et al., 2000; Ozer et al., 2003). These factors appear to play an important role in PTSS severity during the early period after adult trauma (Brewin et al., 2000; Ozer et al., 2003). In contrast, ACEs may not play an important role in acute post-traumatic PTSS due to more prevalent influences of factors like emotional and dissociative responses to the trauma. This may partly explain why there was no overall difference in PCL scores across groups at post-trauma 2 weeks. Other factors may also contribute to early PTSS, including trait anxiety (Suliman et al., 2013) and type of trauma (Geoffrion et al., 2020), with exposure to actual or threatened serious injury, sexual assault, and near death being among the most common causes of PTSS (Geoffrion et al., 2020). Further studies of interactions of factors for PTSS vulnerability are needed.

Early post-trauma emotional and dissociative responses that contribute to PTSS can decrease over time (Charuvastra and Cloitre, 2008; Thompson-Hollands et al., 2021). This raises a possibility that ACEs, and other factors, play a larger role in regulating PTSS at later post-trauma periods. Our results provide evidence for significant differences in CAPS scores across the three groups at post-trauma 3 months. Compared to the non-ACEs and the Sch-ACEs groups, the survivors with preschool ACEs had the highest CAPS scores at post-trauma 3 months, suggesting that preschool ACEs were more associated with severe PTSS during post-trauma months. This is consistent with results from a study on the influence of age of occurrence of ACEs on PTSS experienced during pregnancy (Atzl et al., 2019), which suggest that earlier, but not later, childhood maltreatment was positively related to PTSD symptoms during pregnancy. This indicates that an early occurrence age of ACEs may increase susceptibly to the development of PTSD after adult trauma.

An explanation from a different perspective examines the possibility that greater PTSD symptoms may be due to the longer cumulative effect of ACEs. In our study, all patients with stress during preschool ages also reported stress during school ages. On the contrary, some patients reported childhood stress only during school ages, and therefore had a lower cumulative amount of reported stress. This thought is consistent with a previous study. The recent study found that high exposure to ACEs at a younger childhood age was significantly associated with high exposure to ACEs at a later childhood age. Furthermore, their study found that persistent exposure to ACEs predicted higher scores on both the Childhood Behavior Checklist and PTSD Reaction Index (Dierkhising et al., 2019). Their results are supportive of our findings.

In contrast, one report from other work found no association between PTSS severity and the occurrence age of ACEs in adult psychiatric patients (Schalinski et al., 2016). A possible explanation for the differences in findings is the age ranges that were studied. The ACEs age ranges in the current study, before age 6 and ages 6–13, are broader than the age ranges in the Schalinski et al. study, which focused on preschool (age 4–5) and pre-adolescence (ages 8–9). Although Schalinski et al. results show no association between the age of occurrence of ACEs and PTSS severity, the differing age ranges between the two studies shine light on the need for further work on this issue.

### Association between ACEs and left and right thalamic volumes

Our results suggest that earlier ACEs are related to smaller thalamic volume at an early post-trauma time in adult trauma survivors. These results are consistent with previous literature that ACEs are associated with abnormal brain development (De Bellis et al., 1999; Andersen et al., 2008), and total brain volume is found to correlate with the age and duration of ACEs (De Bellis et al., 1999). Childhood physical or sexual abuse is associated with a diminished size of the corpus callosum (De Bellis et al., 1999), reduced hippocampus volume (Bremner et al., 1997), and gray matter alterations in the frontal cortex (De Bellis et al., 2002). Recent work also suggests ACEs may impair thalamic function (Yoshii, 2021) and negatively affect thalamic volumes (Duarte et al., 2016).

Developmental studies report that the brain reaches nearly 90% of adult brain volume by age six (Reiss et al., 1996; Iwasaki et al., 1997; Courchesne et al., 2000; Paus et al., 2001; Lenroot and Giedd, 2006; Stiles and Jernigan, 2010). Furthermore, one study found that the thalamus continues to increase in volume in healthy children age 6–10 (Muftuler et al., 2011). These results suggest that the age ranges in the current study include a time window of major thalamic growth and development; thus, adverse events during this window may have critical impacts on thalamic volume.

To the best of our knowledge, the current study is the first to analyze adult early post-trauma thalamic volumes from the perspective of the occurrence age of ACEs. Our results reveal smaller left and right thalamic volumes when ACEs began before age six when compared to ACEs occurring between the ages of 6–13. Similar to the results regarding the association between an early age of ACEs and PTSD development, the association between early ACEs and thalamic volume could also be partly explained by a longer duration of the ACEs. One animal study showed that a single prolonged stress can result in smaller thalamic volumes (Yoshii et al., 2017). Thus, besides early occurrence, preschool ACEs may also have a longer duration influence on thalamic volumes. The current correlation design does not distinguish if the reported difference in thalamic volume among the study groups was produced prior to and/or after the adult trauma. Future work using a pre-post trauma brain imaging design would be very useful.

### Thalamic volume moderation of PTSS progression from 2 weeks to 3 months after adult trauma

Our results suggest that the positive relationship between PCL scores at 2 weeks and CAPS scores at 3 months after adult trauma largely exists for subjects with smaller thalamic volumes, rather than subjects with larger thalamic volumes. This indicated thalamic volumes contribute to PTSS symptom trajectories during the weeks-to-months after adult trauma.

Previous work suggests that smaller left thalamic volumes are associated with severe post-trauma re-experiencing symptoms (Shucard et al., 2012). In addition, one study found decreased thalamic gray matter volume was associated with higher PCL scores (Steuber et al., 2021). Furthermore, functional alterations in the thalamus have been linked to stress-related processing changes, including incorrect integration of trauma-related sensory inputs (Brewin, 2001), inappropriate attention processing (Suvak and Barrett, 2011), and over-consolidation of traumatic memory (Brewin, 2001; Suvak and Barrett, 2011). It has also been proposed that reduced thalamic activity may be related to reduced ability to cope with stress (Zhang et al., 2019). This raises a possibility that thalamic structural and functional changes may impair sensory processing and/or the ability to cope with stress. Survivors with smaller thalamic volumes and reduced coping ability could contribute to persistent PTSS over post-trauma weeks-to-months and ultimately to PTSD development. Consistent with this possibility, the present results suggest that, together with other factors, moderating effects of small thalamic volumes on early post-trauma PTSS progression may contribute to PTSD development. Given an influence of age of occurrence of ACEs, early ACEs may have a stronger impact on adult stress responses and, increase vulnerability to PTSD symptom development after adult trauma.

### Limitations

This study has several limitations. The sampled ED trauma survivors had high stress and pain at the time of trauma, thus the current results may more represent trauma survivors with higher risks for PTSD. Many factors impact ACEs, thalamic volume, stress, and PTSD symptoms, and not all are assessed or controlled in the present study. Furthermore, in the two ACEs groups, there are more than twice as many individuals with physical assault trauma compared to the non-ACEs group. Although this is not a statistically significant difference, the lack of significance may be due to the small group sizes. Therefore, recent traumatic physical assault may be a contributing factor to the differences between ACEs and non-ACEs groups, although it does not appear to play a role in the differences between the two ACEs groups. The employed CARSS survey evaluates stress levels for preschool and school ages, but does not identify the types of aversive events, or causes of stress. We acknowledge that the types and causes of adverse childhood events need future attention. We acknowledge that further work on specific ages of occurrence of particular childhood trauma and stress using a more direct age definition than possible with our retrospective approach would be useful. With regard to ACEs questionnaires completed by participants, recall bias is a possibility. For example, recent studies reported that retrospective recall of ACEs may be influenced by stress or other states of the respondent at the time of the retrospective report. In other words, recency factor effects may bias recall of ACEs severity (Dierkhising et al., 2019; Widom, 2019). To reduce this, only the survivors with consistent CTQ and CARSS scores, which clearly presented either positive ACEs or no ACEs, were included. Demographic variation effects involving subjects’ ethnicity/race were not analyzed. It has been known that ACEs are associated with mental health disorders, including depression, anxiety, and substance addictions which can be comorbid factors for PTSD; however, these factors were not assessed in this study. Finally, future work that can demonstrate causal relationships between age, thalamic volume, and symptoms is needed.

## Conclusion

Early ACEs were associated with smaller thalamic volumes, which, in turn, appeared to moderate PTSS severity and PTSD development over weeks to months after adult trauma. This suggests that age of occurrence of ACEs may be a risk factor for maldevelopment of thalamic volume and persistent PTSD symptom after adult trauma. This points to the need for further work to test this provocative possibility.

## Data availability statement

The datasets presented in this study can be found in online repositories. The names of the repository/repositories and accession number(s) can be found below: https://ndar.nih.gov/edit_collection.html?id=2541.

## Ethics statement

The studies involving human participants were reviewed and approved by the Institutional Review Board University of Toledo. The patients/participants provided their written informed consent to participate in this study.

## Author contributions

NH: literature review, data analysis, and manuscript writing. C-HS: sMRI brain image collection and processing and statistic consultant. AC: sMRI brain image collection and processing and subject recruitment. TL: sMRI brain image clinical inspection. SG: supervise subject enrollment in emergency room. JW: study design and manuscript editing. XW: study PI and study design. HX: subject recruitment, data collection, data analysis and manuscript finalization. All authors contributed to the article and approved the submitted version.

## Footnotes

1
https://surfer.nmr.mgh.harvard.edu
